# ADAGE-Based Integration of Publicly Available *Pseudomonas aeruginosa* Gene Expression Data with Denoising Autoencoders Illuminates Microbe-Host Interactions

**DOI:** 10.1128/mSystems.00025-15

**Published:** 2016-01-19

**Authors:** Jie Tan, John H. Hammond, Deborah A. Hogan, Casey S. Greene

**Affiliations:** a Department of Genetics, Geisel School of Medicine at Dartmouth, Hanover, New Hampshire, USA; b Department of Microbiology and Immunology, Geisel School of Medicine at Dartmouth, Hanover, New Hampshire, USA; c Department of Systems Pharmacology and Translational Therapeutics, Perelman School of Medicine, University of Pennsylvania, Philadelphia, Pennsylvania, USA; Argonne National Laboratory

**Keywords:** genomics, denoising autoencoders, bioinformatics, gene expression, data integration

## Abstract

The increasing number of genome-wide assays of gene expression available from public databases presents opportunities for computational methods that facilitate hypothesis generation and biological interpretation of these data. We present an unsupervised machine learning approach, ADAGE (*a*nalysis using *d*enoising *a*utoencoders of *g*ene *e*xpression), and apply it to the publicly available gene expression data compendium for *Pseudomonas aeruginosa*. In this approach, the machine-learned ADAGE model contained 50 nodes which we predicted would correspond to gene expression patterns across the gene expression compendium. While no biological knowledge was used during model construction, cooperonic genes had similar weights across nodes, and genes with similar weights across nodes were significantly more likely to share KEGG pathways. By analyzing newly generated and previously published microarray and transcriptome sequencing data, the ADAGE model identified differences between strains, modeled the cellular response to low oxygen, and predicted the involvement of biological processes based on low-level gene expression differences. ADAGE compared favorably with traditional principal component analysis and independent component analysis approaches in its ability to extract validated patterns, and based on our analyses, we propose that these approaches differ in the types of patterns they preferentially identify. We provide the ADAGE model with analysis of all publicly available *P. aeruginosa* GeneChip experiments and open source code for use with other species and settings. Extraction of consistent patterns across large-scale collections of genomic data using methods like ADAGE provides the opportunity to identify general principles and biologically important patterns in microbial biology. This approach will be particularly useful in less-well-studied microbial species.

**IMPORTANCE** The quantity and breadth of genome-scale data sets that examine RNA expression in diverse bacterial and eukaryotic species are increasing more rapidly than for curated knowledge. Our ADAGE method integrates such data without requiring gene function, gene pathway, or experiment labeling, making practical its application to any large gene expression compendium. We built a *Pseudomonas aeruginosa* ADAGE model from a diverse set of publicly available experiments without any prespecified biological knowledge, and this model was accurate and predictive. We provide ADAGE results for the complete *P. aeruginosa* GeneChip compendium for use by researchers studying *P. aeruginosa* and source code that facilitates ADAGE’s application to other species and data types.

**Author Video**: An author video summary of this article is available.

## Abstract (precis)

The quantity and breadth of genome-scale data sets that examine RNA expression in diverse bacterial and eukaryotic species are increasing more rapidly than for curated knowledge. Our ADAGE method integrates such data without requiring gene function, gene pathway, or experiment labeling, making practical its application to any large gene expression compendium. We built a *Pseudomonas aeruginosa* ADAGE model from a diverse set of publicly available experiments without any prespecified biological knowledge, and this model was accurate and predictive. We provide ADAGE results for the complete *P. aeruginosa* GeneChip compendium for use by researchers studying *P. aeruginosa* and source code that facilitates ADAGE’s application to other species and data types.

## INTRODUCTION

Modern biomedical research routinely generates rich data sets measuring genome-wide gene expression, and advances in sequencing technology have dramatically reduced the cost and increased the use of genome-wide assays of gene expression (1–7). Many methods exist to identify important signals from data generated within a single experiment, e.g., clustering (8–10) or differential expression analysis (11), but integrative analyses across many data sets are more challenging, particularly for microbial systems in which many different conditions are assessed. In well-studied species, integrative analyses of gene expression often employ supervised methods that leverage prior knowledge to extract information from noisy data present in large publicly available data sets (12–14). In less-well-studied organisms, the task of large-scale gene expression analysis is more challenging (15, 16) due to limited information about gene function and the absence of prior knowledge about the organism’s biology. As the accumulation of data exceeds curation, particularly in nonmodel organisms, new unbiased approaches to reveal biological patterns are required.

Deep-learning algorithms have transformed how underlying explanatory factors are extracted from diverse large-scale unlabeled data sets (17). Denoising autoencoders (DAs) (18), examples of one form of deep learning, extract important signals and construct representative features, referred to as nodes, by training models to remove noise that is intentionally added to input data. DAs successfully recognize handwritten digits (18), spoken words (19), and the sentiment of Amazon reviews (20). Because the DA’s learning objective is defined entirely by the data, this algorithm can extract meaningful features without requiring prior knowledge, which makes DAs well-suited to the challenge of data integration for nonmodel organisms.

Here, we report the development of an approach based on *a*nalysis using *d*enoising *a*utoencoders of *g*ene *e*xpression (ADAGE), which is capable of integrating diverse gene expression data to aid in the interpretation of existing and new experiments. Using an unsupervised machine learning approach, the community-wide *Pseudomonas aeruginosa* gene expression data were integrated to create an ADAGE model that captures patterns that correspond to biological states or processes in gene expression data. In our analysis, each data set was interpreted in terms of the activity of 50 distinct nodes, with each node being influenced by different sets of genes. In positive-control analyses, we found that cooperonic genes were preferentially linked to common nodes and that genes with similar KEGG functions had similar gene-node relationships across the model. More interestingly, ADAGE extracts certain nodes representing recognizable identities with predictive values. Additionally, we show that ADAGE is capable of revealing subtle but biologically meaningful signals within existing data sets. We compared ADAGE with existing popular feature construction approaches, including principal component analysis (PCA) and independent component analysis (ICA). The features captured by ADAGE were not fully extracted by either PCA or ICA. The unsupervised ADAGE approach can be applied to any large publicly available gene expression compendium or newly generated gene expression data to characterize genomic and transcriptional features.

## RESULTS

### Construction of an ADAGE model for *P. aeruginosa.*

**FIG 1:**
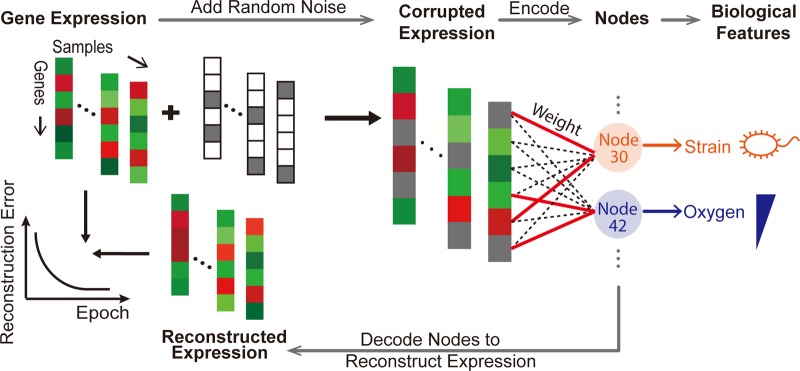
ADAGE model example. For one sample in the expression compendium (one column in the figure with red or green colors, representing expression values of various genes), random noise is first added to the expression value. The corrupted expression values are then encoded into 50 nodes through a gene-to-node weight matrix, which connects each gene to each node. A red solid line represents a high-weight relationship between a gene and a node, indicating that the gene has a stronger influence on the node’s activity than other genes (connected by black dotted lines). Node activities derived from this sample are decoded back into reconstructed expression values through the same weight matrix. Samples in the compendium are trained through the encoding and decoding steps with the goal of minimizing differences between initial expression values and reconstructed expression values. The resulting ADAGE model constructs nodes from genomic measurements that can be interpreted as biologically meaningful features, such as genome divergence among strains and transcriptional responses to oxygen abundance.

To build the ADAGE model for the analysis of *P. aeruginosa* gene expression, we focused on expression profiling performed using Affymetrix GeneChips, because of the uniform gene nomenclature. All *P. aeruginosa* GeneChip expression data were downloaded from the ArrayExpress database (21), and this resulted in a compendium of 950 arrays from 109 experiments (see File S1 in the supplemental material). We constructed an ADAGE-based model from the compendium by first adding random noise to the input data and then training a neural network with hidden nodes that were able to remove added noise to reconstruct the initial data (Fig. 1; see Materials and Methods for further details). The process of adding noise improves the robustness of constructed features and consequently the resulting models (22, 23). The resultant network was designed to contain 50 nodes, and within each node all *P. aeruginosa* strain PAO1 genes were assigned weights that reflected the contribution of each gene to the activity of each node (see File S2 in the supplemental material for weight vectors). A model with 50 nodes was chosen to balance reconstruction error with the need to manually interpret the ADAGE model, and our subsequent analyses demonstrated that networks of this size are capable of adequately extracting major global transcriptional patterns (Fig. 1).

10.1128/mSystems.00025-15.4 File S1 The *Pseudomonas aeruginosa* Gene Expression Compendium. This file covers all samples in publically available data sets collected before 2 February 2014. They were combined using the rma function, with quantile normalization provided in Bioconductor’s affy R package. Only transcripts with PA IDs were maintained. Download File S1, TXT file, 65.1 MB. Copyright © 2016 Tan et al. 2016 Tan et al. This content is distributed under the terms of the Creative Commons Attribution 4.0 International license.

10.1128/mSystems.00025-15.5 File S2 ADAGE weight matrix. This file contains each gene’s weight contribution to each node. Download File S2, XLS file, 5.06 MB. Copyright © 2016 Tan et al. 2016 Tan et al. This content is distributed under the terms of the Creative Commons Attribution 4.0 International license.

**FIG 2:**
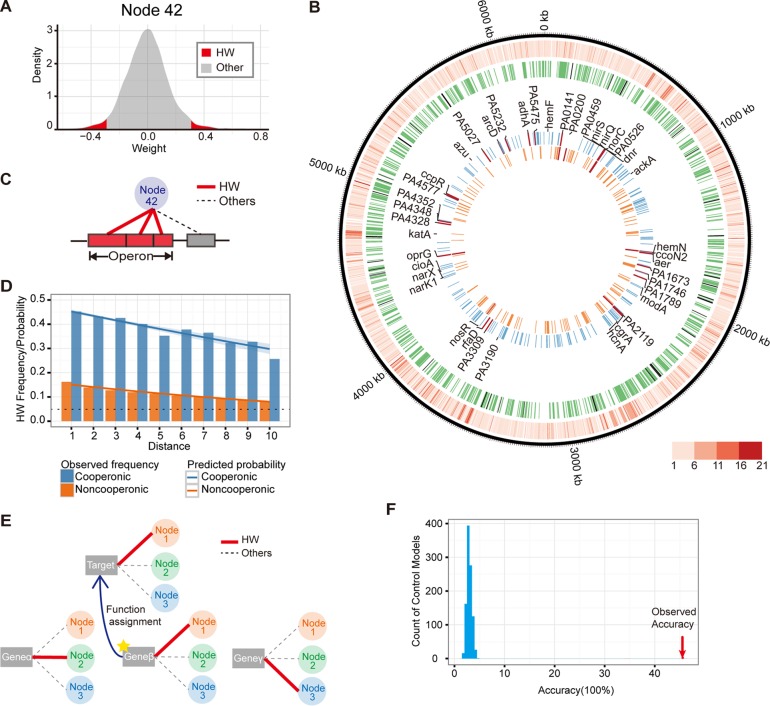
ADAGE weights reflect a gene’s common regulatory and process features. (A) HW genes defined in ADAGE. The distribution of edge weights that connect genes to each node, e.g., node 42 shown here, is approximately normal. HW genes in a node were defined as genes whose weights were more than 2 standard deviations from the mean (shown in red). (B) The contributions of individual genes and operons to the ADAGE model. The outermost ring shows those genes that are HW within at least one node. The color intensity reflects how many nodes (ranging from 1 to 21) a gene was connected to at a high weight. The second-to-outermost circle shows operons that were significantly associated with at least one node (green bars) and those that were not significantly associated with any of the 50 nodes (black bars). The inner two circles represent the HW genes in nodes 42 and 30 (outside to inside). The labeled genes are those identified by Trunk et al. (37) and Jackson et al. (36) as regulated by Anr and they are colored red among HW genes of nodes 30 or 42. The HW genes in node 42 overlapped extensively with Anr-regulated genes, which suggested that node 42 captured the regulatory signature of Anr. (C and D) ADAGE captured principles of bacterial genome organization. (C) In the bacterial genome, genes are arranged into operons, which the ADAGE model recognizes by connecting cooperonic genes to a shared node. (D) The regulatory role of genome positioning in *P. aeruginosa* was captured by ADAGE. A logistic regression analysis revealed that cooperonic genes (blue line shows model data; bars show observed data) were more likely to be co-HW genes than noncooperonic genes (red). As the number of genes between two genes on the chromosome increased, they were less likely to be co-HW. The black dotted line represents the background frequency of HW genes across all nodes. (E and F) ADAGE captured gene functional features. (E) We found the closest neighbor of a target gene based on the Euclidean distance between the weight vectors connecting each gene to 50 nodes and assigned the closest neighbor gene’s function to the target gene. (F) The accuracy of gene function assignment using the ADAGE model (45%, red arrow) was much higher than the accuracies achieved with 1,000 randomly permuted control models (distribution shown in blue). Here, we considered a function assignment positive if one or more functions assigned by the closest neighbor gene matched the target gene’s annotations.

All genes were connected to each node by a weight vector, and the contributions, or gene weights, within a node were distributed symmetrically and approximately centered at 0. These weights approximately resembled a normal distribution in which a small proportion of genes provided high positive or high negative weights to that node (Fig. 2A). We refer to genes that were outside 2 standard deviations as high-weight (HW) genes for that node (Fig. 2A, red regions). In the ADAGE model, 4,029 genes (72.6% of the *P. aeruginosa* genome) were HW in at least one node (Fig. 2B, outermost ring; see also File S3 in the supplemental material), and 229 (4.1%) genes were HW in 10 or more nodes. It is important to convey that nodes differed in terms of the identities of the HW genes. The innermost rings shown in Fig. 2B illustrate the HW genes in two example nodes (nodes 42 and 30, respectively, from outside to inside), which we identified as representative of anaerobic response and strain specificity, as discussed in more detail below.

10.1128/mSystems.00025-15.6 File S3 HW genes for each node and their corresponding weights in that node. Each column in this file either stores the list of high-weight genes that are outside 2 standard deviations in the node’s weight distribution or stores the list of genes’ corresponding weights. High-weight gene columns are ordered by weight. Download File S3, XLS file, 0.5 MB. Copyright © 2016 Tan et al. 2016 Tan et al. This content is distributed under the terms of the Creative Commons Attribution 4.0 International license.

### Operonic comembership and spatial proximity reflect gene-node relationships.

Bacterial operons, by definition, contain genes that are coexpressed, though genes within an operon may be transcribed by multiple promoters. To determine if genes within operons shared similar node relationships, gene set enrichment analysis (GSEA) of ADAGE weights using operon annotations from DOOR (24) were performed, and the results showed that, in total, 92.9% of cooperonic genes were significantly associated with at least one common node (Fig. 2B, second-to-outermost circle; operons significantly associated with at least one node are shown in green, and unassociated operons are in black). The operons significantly associated with each node are shown in File S4 in the supplemental material. As an extension of the above analysis of operon-node relationships, we also predicted that cooperonic genes would be HW to the same nodes (Fig. 2C). To test this prediction, we fit a logistic regression model that predicted whether a gene was likely to be HW in a given node based on whether cooperonic genes were also HW to that node. As predicted, genes cooperonic with an HW gene had a 4.6-times-higher odds of being HW in the same node. In addition, we determined if genes were more likely to be HW to the same node if they were spatially proximal (e.g., a small number of intervening genes) even if they were not cooperonic. Again, as expected, the odds of two adjacent genes being HW to the same node were higher for cooperonic genes than for noncooperonic genes (Fig. 2D). Interestingly, for both cooperonic and noncooperonic genes, every additional intervening gene between two genes decreased the odds of them being HW to the same node by a factor of 0.9 (Fig. 2D), indicating links between proximity and coexpression. This trend could reflect that genes within a pathway are often physically close or that there are other regional factors that affect local gene expression in a coordinated way.

10.1128/mSystems.00025-15.7 File S4 Operon-node association in the ADAGE model. Significance for the association of cooperonic genes with a given node, indicating that cooperonic genes are likely to have similar high weights in a node. Only significant node-operon relationships are listed. Association with a node in this analysis does not imply that the genes in the operon are necessarily HW (outside 2 standard deviations) in that node. Download File S4, XLS file, 0.4 MB. Copyright © 2016 Tan et al. 2016 Tan et al. This content is distributed under the terms of the Creative Commons Attribution 4.0 International license.

10.1128/mSystems.00025-15.8 File S5 ADAGE node activities for each sample. The node activity is calculated as the dot product of the sample’s expression vector and the node’s weight vector. Download File S5, XLS file, 0.25 MB. Copyright © 2016 Tan et al. 2016 Tan et al. This content is distributed under the terms of the Creative Commons Attribution 4.0 International license.

### Genes within a common KEGG pathway share node relationships.

To further test the biological relevance of the ADAGE model, we predicted that genes involved in the same pathway would have similar gene-node relationships. We tested this prediction using *post hoc* analysis of the ADAGE model via the Kyoto Encyclopedia of Genes and Genomes (KEGG) database (25). To do so, we employed a straightforward algorithm in which a target gene was assigned the KEGG function of its closest “neighbor” based on ADAGE weights; the neighbors for each gene were determined by calculating the Euclidean distance between each gene’s connections to all nodes for every gene pair (Fig. 2E). If a gene’s predicted KEGG functions based on the functions of its closest ADAGE model neighbor matched at least one of its actual KEGG annotations, it was considered positive. If no KEGG annotations matched, it was considered negative. We used this approach because our goal was to evaluate the model itself. Though more complex techniques would be likely to provide superior predictions of function, they would not be as useful for direct evaluation of the underlying model. We observed a high accuracy of gene-function assignment (45%) with the ADAGE model. As a control, the identical algorithm was applied to 1,000 control models, where we randomly permuted gene identifiers. In these tests, the mean accuracy was 3% with no permuted model achieving greater than 5% accuracy (Fig. 2F). We also evaluated a more stringent definition of correct assignment that requires all predicted and annotated functions to match and observed consistent results. In this analysis, 34% of gene-function assignments were correct when the ADAGE model was used, while less than 3% of assignments were correct when randomly generated models were used. These analyses suggest that ADAGE-based grouping of genes into nodes based on expression across the *Pseudomonas* GeneChip compendium identified biologically relevant relationships between genes.

### ADAGE recognizes genomic differences between strains.

**FIG 3:**
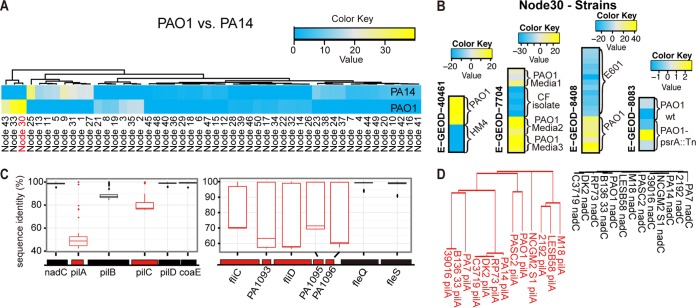
ADAGE extracted features that represented sequence differences between strains. (A) ADAGE node activity heat map analysis of the genome hybridization of *P. aeruginosa* strains PA14 and PAO1 to Affymetrix *Pseudomonas* GeneChips. The heat map shows the ADAGE activity differences of each node in each strain when the genome hybridization data were analyzed. The activity values of node 30 most strongly differentiated strains PAO1 and PA14. (B) Analysis of mean centered node 30 activity values clearly distinguished strain PAO1 from other *P. aeruginosa* strains (HM4, E601) or uncharacterized CF strains in RNA expression experiments. For each heat map, the complete range of mean-centered node activities was used to generate the color range. The range of activity values in experiments that compared strains was at least 40 (−20 to 20), whereas the range of node 30 activities was much less in a data set in which only PAO1 or its derivatives were analyzed. (C) Pairwise percent sequence identities between orthologous genes from 13 *P. aeruginosa* strains. Two operons that contain the top 5 HW genes in node 30 were analyzed. HW genes are shown in red. (D) Phylogenetic trees of *pilA* (the most HW gene) and *nadC* (pilA’s immediate neighbor). The two trees share the same distance per branch length unit, and the longer branch represents more genetic changes.

We predicted that the HW genes within a node were likely grouped together because they were related in their expression patterns across many data sets in the compendium. Visual inspection of the lists of HW genes (see File S3 in the supplemental material) revealed that several nodes contained genes that are known to vary between strains (those for lipopolysaccharide [LPS], flagellin, pili, etc.) (26). Because the *P. aeruginosa* compendium includes results from experiments performed on different *P. aeruginosa* strains, we sought to determine if strain-specific signatures were represented in the ADAGE model. To do this, we isolated DNA from two well-studied strains of *P. aeruginosa*, PAO1 (27) and PA14 (28), and performed a DNA hybridization experiment. Hybridization to the *Pseudomonas* GeneChip yielded a profile in which a small number of ADAGE nodes were highly differentially active (Fig. 3A). In the three most differentially active nodes (30, 33, and 25, in order of difference magnitude), we found many genes that are known to associate with strain-to-strain differences within the species. We focused our further analyses on node 30, which exhibited the largest difference in activity between PA14 and PAO1. A functional enrichment analysis by use of Gene Ontology (GO) (29) and KEGG (25) terms found that the HW genes in node 30 included those associated with the surface-exposed portions of type IV pili (*pilA*, *pilC*, *pilV*, *pilW*, *pilY1*, and *pilY2*) and the flagellum (*flgK*, *flgL*, *fliC*, and *fliD*) (see Table S1 in the supplemental material). Importantly, the ADAGE model precisely identified specific genes within pilus and flagellum gene clusters as contributing strongly to the identity of node 30. For example, two genes involved in pilus biosynthesis, *pilA* and *pilC*, were among the HW genes in node 30, while the adjacent genes *pilB* and *pilD* were not (Fig. 3C, gene relationships). We performed an alignment and pairwise comparison of the *pilABCD* coding sequences from 13 sequenced *P. aeruginosa* strains. This analysis revealed that *pilA* and *pilC* were strikingly more divergent than either *pilB* or *pilD* or than other adjacent genes *nadC* and *coaE* (Fig. 3C and D). In fact, *pilA* had the highest weight in node 30 and was the most divergent gene of those analyzed (Fig. 3C and D). A similar trend was observed for the flagellum-associated genes: the five HW flagellar genes in node 30 varied in sequence across strains (Fig. 3C), but two adjacent genes, *fleQ* and *fleS*, were highly conserved and were not HW in node 30 (Fig. 3C). In further support of the hypothesis that the activity of node 30 identifies strain differences, this node contains other strain-specific genes, including those involved in LPS biosynthesis (*wbpA*, *wbpB*, *wbpD*, *wbpE*, *wbpG*, *wbpH*, *wbpI*, *wbpJ*, *wbpK*, *wbpL*, and *wzz*), a putative type I restriction/modification system (loci PA2730 to PA2736), and pyoverdine biosynthesis. The genes encoding bacteriophage Pf4 (PA0717 to PA0734), which is only found in certain strains of *P. aeruginosa* (30), as well as the highly strain-specific R-pyocins (PA0621 to PA0648) (31), were also among the HW genes in node 30. HW genes in the strain-differentiating nodes also included genes that were either unique to PAO1 (PA3501 to PA3504) or only found in a subset of the *P. aeruginosa* strains with published genomes, such as PA0202 to PA206, which encode putative transporter genes.

10.1128/mSystems.00025-15.9 Table S1  Top 10 associated GO terms and KEGG pathways for each node mentioned in our report. Download Table S1, DOCX file, 0.1 MB. Copyright © 2016 Tan et al. 2016 Tan et al. This content is distributed under the terms of the Creative Commons Attribution 4.0 International license.

To determine if node 30 activity differed in gene expression experiments that made comparisons across strains, we identified published experiments within the *P. aeruginosa* GeneChip compendium in which multiple strains were measured. This analysis found that node 30 was indeed differentially active in experiments that included the comparison of different *P. aeruginosa* strains (Fig. 3B, data sets E-GEOD-40461, E-GEOD-7704 [32], and E-GEOD-8408 [33]). In data set E-GEOD-7704, node 30 clearly indicated the differences between lab strain PAO1 and clinical cystic fibrosis (CF) strains. In contrast, node 30 activity did not differ in a control experiment using the same strain (E-GEOD-8083 [34]) (Fig. 3B; the small range in the color key indicates no difference among samples from the same strain). In the Discussion, we address the need for a community-wide allele nomenclature for variable genes to support these types of analyses across data sets and strains when presence/absence is not the readout for strain variation.

### ADAGE node activities reflect transcriptional responses.

To further test whether the node-based ADAGE model identified biological states in gene expression data, we analyzed experiments in which *P. aeruginosa* wild-type cells were compared to cells that lacked the transcription factor Anr. Anr is active in low-oxygen environments and regulates the cellular response to oxygen limitation (35) and other virulence-related processes (36). We found that node 42 was most significantly enriched for HW genes known to be regulated by Anr (false-discovery rate [FDR] *q* value, 4.24e−31). The Anr-regulated genes used in this analysis (36, 37) are listed in Table S2 in the supplemental material. To investigate whether ADAGE could reliably extract an Anr signal from the compendium, we built 100 ADAGE models with different random seeds and repeated the enrichment test. Across the 100 models, 80 contained nodes as significant as or more significant than node 42 in the current model. All 100 models contained a node significantly associated with Anr targets. This indicated that ADAGE robustly identified strong transcriptional patterns across independent applications of the algorithm.

10.1128/mSystems.00025-15.10 Table S2  Anr-regulated gene list used to identify significantly enriched nodes for genes regulated by Anr. Download Table S2, DOCX file, 0.06 MB. Copyright © 2016 Tan et al. 2016 Tan et al. This content is distributed under the terms of the Creative Commons Attribution 4.0 International license.

**FIG 4:**
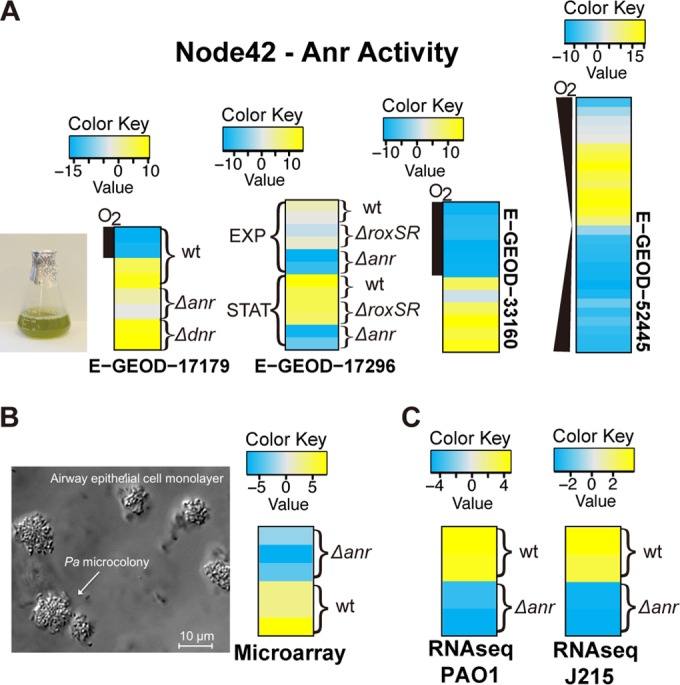
Node 42 reflected Anr activity in both existing and new experiments. (A) Mean-centered activity heat maps of node 42 for four data sets for cells grown in liquid culture. We examined the effects *anr* mutants or altered oxygen levels. In E-GEOD-17179 and E-GEOD-17296, the low activity value (blue or gray under anaerobic conditions) of node 42 corresponded to *anr* deletion. Similar effects were not observed for *dnr* or *roxSR* mutants. In E-GEOD-17179 and E-GEOD-33160, the oxygen bar represents whether or not *P. aeruginosa* is in an aerobic or anaerobic environment. E-GEOD-52445 is a high-time-resolution experiment transiting from high oxygen tension to low oxygen tension, which was then reversed. The activity value of node 42 was negatively correlated to the oxygen abundance in the microbes’ living environment. These results reflected Anr’s role as a transcriptional activator in the context of low environmental oxygen. (B) We performed a validation experiment in which *P. aeruginosa* wild-type and *anr* mutant cells were grown as microcolonies on CF airway epithelial cell monolayers. Although the validation set presents a distinct experimental system from the liquid culture-based experiments in panel A, node 42 activity reflected the *anr* mutant, indicating the robustness of the ADAGE model. (C) We assessed the ADAGE model for two RNA-Seq data sets, both with *anr* mutant and wild-type *P. aeruginosa*. Node 42 again differed (FDR *q* value, 0.05 in PAO1 strain and 0.10 in J215 strain) when the wild-type and *anr* mutant were compared in both strains.

We examined the activity of node 42 in two data sets that compared wild-type and Δ*anr* strains (Fig. 4A, E-GEOD-17179 [37] and E-GEOD-17296 [38]). Node 42 showed low activity (blue) in the *anr* mutant, even under low-oxygen conditions that would otherwise be expected to activate Anr (gray). In addition, we also evaluated the activity of node 42 in data sets where the responses to different oxygen concentrations were compared and found that again, the activity of node 42 was modulated by oxygen availability (Fig. 4A, E-GEOD-33160 [39] and E-GEOD-52445 [40]). E-GEOD-52445 is a high-time-resolution analysis of *P. aeruginosa* transiting from high oxygen tension to low oxygen tension and then the reverse (40). Node 42 activity values gradually increased as oxygen levels decreased in data set E-GEOD-52445, and the restoration of oxygen was concomitant with a striking decrease in node 42 activity. The activity pattern for node 42 in all of the data sets in the compendium can be viewed at http://greenelab.github.io/Paeruginosa-da/.

To evaluate the ADAGE model’s robustness in terms of the relationship between Anr activity and the activity of node 42, we performed an independent experiment in which *P. aeruginosa* wild-type and Δ*anr* mutant cells were grown in association with CF bronchial epithelial (CFBE) cell monolayers (Fig. 4B). This experiment differed from the majority of those in the *P. aeruginosa* gene expression compendium, as the bacteria were grown as planktonic cultures. ADAGE analysis of the genome-wide expression measurements for mRNAs from both the *P. aeruginosa* wild type and Δ*anr* strain, with three biological replicates per strain, confirmed that node 42 reflected the absence of *anr*. This demonstrated that ADAGE not only described the patterns in data within the array experiments used to build the model, but also was able to detect these patterns in experiments performed in environments not well-represented in the training data set. ADAGE analysis also revealed that under this condition, the deletion of *anr* significantly impacted 19 other nodes (*t* test with an FDR threshold of 0.05), consistent with the observation that Anr impacts the direct and indirect expression of many pathways in surface-associated cells (41).

Since *Pseudomonas* GeneChip data were used to build the ADAGE model and the validation experiments above employed additional microarray data, we next assessed the use of ADAGE for interpretation of transcriptome sequencing (RNA-Seq) data. We applied the TDM (training distribution matching) method described by Thompson et al. (42) to normalize RNA-Seq data to a comparable range before ADAGE analysis. Using a recently published data set in which gene expression was analyzed in two strains and their Anr derivatives were grown as colonies in a 1% oxygen atmosphere (41), we found that ADAGE’s node 42 differed (FDR *q* value, 0.05 in the PAO1 strain and 0.10 in the J215 strain) when the wild-type and Δ*anr* mutant strains were compared (Fig. 4C). This demonstrated that ADAGE can also be used to interpret RNA-Seq data, and our goal is for future iterations of ADAGE to be built using both microarray and RNA-Seq data.

### ADAGE reveals subtle patterns contained in existing experiments.

**FIG 5:**
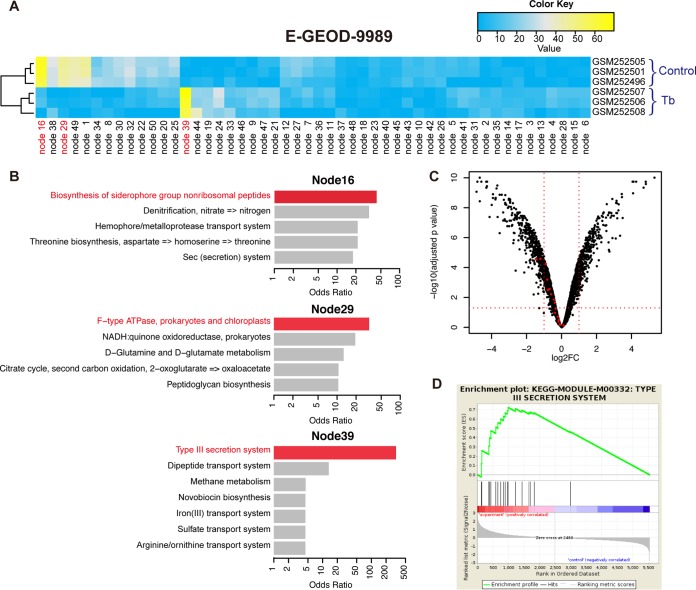
Reanalysis of an existing experiment using ADAGE. (A) Node activity heat map for the data set E-GEOD-9989. Nodes 39, 16, and 29 are the three nodes that most strongly differentiated control samples from those challenged with tobramycin. (B) KEGG pathway analysis of differentially active nodes. The top 5 most enriched KEGG pathways (including ties) based on odds ratios are shown for each node. Nodes 39, 16, and 29 each represent a cellular process being influenced in the experiment (in red), especially node 39, which captures the subtle change in the T3SS pathway. (C) Volcano plot showing differentially expressed genes in E-GEOD-9989. The horizontal dotted red line indicates an adjusted *P* value cutoff of 0.05, and the two vertical lines correspond to log_2_ fold changes of −1 and 1. Genes in the KEGG T3SS pathway (KEGG-Module-M00332) are highlighted in red. Although only two genes passed both the significance cutoff and fold change cutoff, genes in the pathway showed consistently lower expression in tobramycin-treated samples. (D) Gene set enrichment analysis of the T3SS pathway. GSEA also captured the consistent differential expression pattern of T3SS, which it ranked as the 12th most significantly enriched pathway.

Using another previously published data set in which *P. aeruginosa* was grown in association with CFBE cells in culture, we demonstrated that the ADAGE model can reveal important patterns associated with low-magnitude gene expression changes. In this analysis, we reexamined the response of *P. aeruginosa* biofilms to challenge with either the antibiotic tobramycin or with the vehicle control for 30 min (E-GEOD-9989 [43]). In this data set, nodes 39, 16, and 29 were most differentially active (Fig. 5A). KEGG pathway enrichment analysis of HW genes in nodes 16 and 29 revealed enrichment of genes involved in siderophore biosynthesis and ATPase activity, respectively (Fig. 5B), and differential expression of genes in pathways upon tobramycin treatment was evident in the array data. Siderophore biosynthetic transcripts and transcripts involved in energy generation were decreased 2- to 29-fold and 3- to 20-fold, respectively, in response to the antibiotic treatment (see Table S4 in Anderson et al. [43]). The node with the most differential activity between tobramycin-treated and untreated cells was node 39 (Fig. 5A), and the HW genes in node 39 were most enriched in genes associated with the type 3 secretion system (T3SS) (Fig. 5B). A standard microarray analysis approach did not detect a strong expression difference in genes involved in the T3SS; among 775 differentially expressed genes (log_2_-fold change, >1; adjusted *P* value, <0.05, after fitting a gene-wise linear model using the limma R package [44]), only 2 out of 18 genes in the T3SS KEGG pathway were differentially expressed (Fig. 5C). However, those authors did observe a difference in T3SS-dependent cytotoxicity, and a subsequent study by the same authors demonstrated that a tobramycin-induced transcript, *mgtE* (see Table S3 in Anderson et al. [43]), repressed *exsA*, the transcriptional activator of the T3SS (45). Thus, we propose that ADAGE can indicate meaningful differences in biology even when the transcriptional difference is low. Small differences in transcript levels associated with a differentially active process can result from expression analysis at a time point that does not capture maximal differential expression, captures differential expression in only a subset of the population, or captures other inherent gene expression properties for transcripts of interest. Because each ADAGE node represents a multigene pattern that was learned from analyzing the entire expression compendium, ADAGE node-based analyses may be particularly able to detect subtle patterns.

The ability to identify meaningful, small but consistent changes in gene expression is also a characteristic of GSEA (46), and GSEA did identify the T3SS pathway as the 12th most significant pathway (Fig. 5D). However, GSEA relies on curation to group genes together in a pathway, while ADAGE directly learns biological features from the data even in the absence of annotation. In well-studied species like *P. aeruginosa*, we were readily able to predict modulation of the T3SS pathway by looking at the identities of the HW genes of node 39. In less-well-studied species, the knowledge regarding genes in differentially active nodes would provide the basis for hypothesizing on the nature of involved pathways based on analysis of coregulated genes, bioinformatics analysis of genes, and targeted genetic and biochemical studies.

### Comparison with PCA and ICA.

PCA (47, 48) and ICA (49–51) are two frequently used feature construction methods in bioinformatics that have also been applied to the analysis of gene expression data. To compare ADAGE-extracted features to those from these other two methods, we performed analyses with each method that was parallel to our ADAGE analysis findings. To compare findings with the 50-node ADAGE-based model, we analyzed the first 50 components in PCA and ICA applied to the expression compendium used to create ADAGE. These components explained >80% of the variance in the compendium. For analysis of the DNA hybridization experiment with PAO1 and PA14 strains, we found PC4/PC5 in PCA and IC26/IC18 in ICA as the two most differentially active components in each approach (see Fig. S1A in the supplemental material). With ADAGE, we analyzed only the most differentially active node, but we evaluated the top two components for PCA and ICA to avoid missing potential signals in the second most differentially active component. While many of the genes that were different in the genome hybridization comparison of strain PA14 and strain PAO1 were HW genes in PC4, PC5, IC26, and IC18, these components failed to accurately capture strain variations in other data sets (see Fig. S1B). In data set E-GEOD-7704, *P. aeruginosa* RNA from the CF sputum was analyzed directly and RNA was harvested from the same set of sputum-derived stains after growth *ex vivo*. While ADAGE node 30 differentiated the samples comprised of clinical strains from samples containing only strain PAO1, the samples containing clinical strains were heterogeneous and not uniformly different from the PAO1 samples with respect to PC4 (see Fig. S1B), indicating that strain differences were not reliably captured by this component. Inspection of HW genes for PC4 revealed that it not only contained strain-specific genes but also a significant number of Anr-regulated genes (FDR *q* value, 1.0e−42), so the differential activity of PC4 is also likely to be influenced by oxygen availability. The increased activity of PC4 in the clinical strain samples from the lung samples in comparison to clinical strain samples grown in the laboratory is consistent with the finding that *P. aeruginosa* is in an oxygen-limited state in the CF lung (52) (see Fig. S1B). Because PCA seeks to find the direction of the largest variance, each component can become a mixture of highly variable processes that are not biologically related. While ICA decomposes data into independent signals and does not have this property, we found that the activities of strain-associated components extracted by ICA were not consistent within the replicates of individual data sets.

10.1128/mSystems.00025-15.1 Figure S1  Comparison of PCA and ICA with ADAGE for the DNA hybridization experiment and extraction of strain-specific features. (A) In ADAGE, node 30 best differentiated PAO1 strain from PA14 strain. PC4 and PC5 in PCA and IC26 and IC18 in ICA were the components that differed the most between two strains. The top two components from PCA and ICA, as opposed to the top one component from ADAGE analysis, were evaluated to give each method the benefit of the doubt. (B) Node 30 from ADAGE clearly separated PAO1 strain from other strains in three independent data sets. PC4, PC5, IC26, and IC18 did not effectively capture the strain variations across the three data sets. Download Figure S1, TIF file, 0.5 MB. Copyright © 2016 Tan et al. 2016 Tan et al. This content is distributed under the terms of the Creative Commons Attribution 4.0 International license.

As in our ADAGE analyses, we also evaluated PCA and ICA components representing oxygen abundance and Anr activity. PC4 (FDR *q* value, 1.0e−42), PC7 (FDR *q* value, 4.5e−46), IC14 (FDR *q* value, 3.2e−20), and IC49 (FDR *q* value, 1.1e−19) were the components most enriched in Anr-regulated genes. While results for these PCA and ICA components were able to identify trends in which Anr-regulated genes were differentially expressed in response to oxygen, the resolution of the Anr signal was notably better when the ADAGE model was used when all of the experiments were considered (see Fig. S2 in the supplemental material). For example, PC7 was comparable to ADAGE node 42 in many experiments, but node 42 outperformed PC7 in the analysis of the Anr microarray data set, because PC7 contains other processes that also changed between the wild type and *anr* mutant strain.

10.1128/mSystems.00025-15.2 Figure S2  Comparison of PCA and ICA with ADAGE for the transcriptional signal of Anr and oxygen abundance. Node 42 from ADAGE robustly reflected Anr activity under various conditions, including aerobic/anaerobic environment, exponential/stationary growth phase, *anr* knockouts grown on CFBE, and *anr* knockouts in strain PAO1 and clinical isolate J215. PC4 did not capture Anr activity in E-GEOD-17179 and E-GEOD-17926. PC7 did not capture *anr* mutant *P. aeruginosa* grown on CFBE (the color key’s small range indicates PC7 cannot differentiate the *anr* mutant from wild type). IC14 and IC49 exhibited non-Anr patterns in multiple experiments. Download Figure S2, TIF file, 0.5 MB. Copyright © 2016 Tan et al. 2016 Tan et al. This content is distributed under the terms of the Creative Commons Attribution 4.0 International license.

Finally, we compared the results from the analysis of E-GEOD-9989, which compared the effects of tobramycin on CFBE-associated *P. aeruginosa*, using ADAGE, PCA, and ICA. The PCA results agreed with those of ADAGE in terms of identifying changes in F-type ATPase-associated genes and transcripts associated with siderophore biosynthesis. In the ADAGE model, the node with the greatest mean difference between tobramycin-treated and untreated cultures (node 39) was most enriched in T3SS-related genes (Fig. 5), and this was consistent with the T3SS-dependent phenotype reported by those authors (43, 45). A similar analysis performed by using PCA and ICA did not indicate changes in the T3SS pathway in the most strongly differentially active components (see Fig. S3 in the supplemental material).

10.1128/mSystems.00025-15.3 Figure S3  Analyzing data set E-GEOD-9989 with PCA and ICA. (A) A heat map representing principal component values shows that PC9, PC1, PC20, and PC4 (in order of absolute difference in mean activities between two conditions) are the PCs most differentially active between samples challenged with tobramycin versus controls. KEGG pathway analysis of the four PCs identified pathways known to be influenced in the data set, such as F-type ATPase, prokaryotes and chloroplasts and biosynthesis of siderophore group nonribosomal peptides, but it did not reveal the subtle changes in the T3SS pathway. (B) A heat map representing independent component values shows that IC11 was strongly differentially active in E-GEOD-9989. However, it also lacked an association with the T3SS pathway. Download Figure S3, TIF file, 0.4 MB. Copyright © 2016 Tan et al. 2016 Tan et al. This content is distributed under the terms of the Creative Commons Attribution 4.0 International license.

In summary, our comparisons using PCA and ICA showed that the biological features extracted by ADAGE were not captured clearly by either of these algorithms. While we expect that PCA and ICA capture certain other biological signals more effectively than ADAGE, our results demonstrate that ADAGE complements these methods by identifying distinct signals.

### ADAGE model availability.

Based on our own usage, we anticipate that our ADAGE model would provide a useful starting point for biological discovery. We have provided two examples of ways to leverage the model. One mode of analysis that we demonstrated begins with the identification of differentially active nodes in a relevant data set (e.g., the genome hybridization experiment in Fig. 3 and the response to tobramycin experiment in Fig. 5). By analyzing HW genes and gene pathways associated with differentially active nodes, we gained a better understanding of differences between samples and revealed the detection of subtle but consistent signals. Heat maps showing differential node activities among samples in each experiment in the *P. aeruginosa* gene expression compendium were generated and are available at http://greenelab.github.io/Paeruginosa-da/.

Another mode of analysis with ADAGE that we demonstrated begins with an investigator-curated list of genes related to a specific process or pathway (e.g., the Anr regulon). By examining nodes associated with the process, researchers may be able to identify novel genes associated with the process as well as other data sets in which the process is different. To facilitate such analyses, the HW genes for each node are provided in File S3 in the supplemental material. The complete ADAGE model, for application to newly performed experiments, is available in File S4 in the supplemental material. Software implementing ADAGE and that performs all of the analyses described in this report is available from https://github.com/greenelab/adage.

## DISCUSSION

Our ADAGE method identifies biological signals (represented by nodes) by intentionally integrating noise into gene expression data prior to the data reconstruction process and model building. Thus, this method is well-suited to the analysis of heterogeneous gene expression data generated in different labs, from experiments with different strains and different growth conditions. The ADAGE methodology differs from other analyses of genome-wide gene expression across large collections, which have generally been performed on homogenous collections (53, 54) or through supervised algorithms that can use known aspects of biology to separate biological signal from noise (12, 55). The *P. aeruginosa* ADAGE model, created without the use of any information on genome structure or gene functions, found that cooperonic genes and adjacent noncooperonic genes were significantly more likely to be involved in similar processes. Furthermore, genes with similar gene-node relationships were much more likely to share a KEGG function than would occur by chance. Because the building phase of ADAGE does not require any prespecified knowledge, we anticipate that ADAGE will find use for organisms with well-curated genomes as well as organisms for which genome curations are lacking. In organisms without curation, the ADAGE model may guide researchers toward gene sets of interest for analysis using additional computational and experimental analyses.

Analysis of existing and newly generated gene expression data using the ADAGE model found expression signatures that correlated with the comparison of different strains and the response to low oxygen. Many genes contribute to the activity scores of each node; thus, node activities can represent patterns resulting from direct or indirect aspects of a given process and may be useful in identifying patterns that are apparent in only a subset of cells in a population. Extracting these subtle but consistent changes from single experiments is difficult or impossible and requires integrative analyses leveraging information from the entire compendium.

Techniques like ADAGE allow multiprocess membership, which means that genes can be assigned to multiple distinct processes simultaneously and these processes can differ in activity independently, as is often the case in biology. In this way, ADAGE is distinct from clustering- or biclustering-based techniques, such as cMonkey (56), which identify subsets of genes coregulated in subsets of experiments. ADAGE is comparable to PCA and ICA, as each gene contributes to nodes via weight, and all nodes have a specific activity in each sample. Through comparison with PCA and ICA, we confirmed that ADAGE extracts signals distinct from these two commonly used feature construction methods. We found that PCA grouped multiple biological sources of variability into top components. Though ICA extracts independent signals, it was not able to capture the same key features of the data captured by ADAGE (strain variation and oxygen abundance). The comparison of ADAGE to PCA and ICA found that ADAGE was superior in grouping replicate samples in the same data set, and this may reflect ADAGE’s strength in dealing with noisy measurements. We propose that both PCA and ADAGE are complementary analytical tools for the unsupervised analysis of large-scale collections of gene expression data. PCA may be preferred for a quick overview of the major sources of variations in a data set, while ADAGE may excel in extracting differentially active biological processes.

As next-generation sequencing facilitates the creation of large gene expression compendia for many organisms, algorithms capable of converting those data into insights about the underlying biological system will be required. In order to capitalize on the wealth of knowledge in large community data sets, communities need to agree upon standardized gene nomenclature for alleles across species. Alternatively, methods to extract allele information for use when two different strains are compared must be developed. In addition, the inclusion of detailed experimental information upon the deposition of gene expression data into public databases will improve the ability for community-wide data to be used by many to understand pathways and processes of interest.

We demonstrated the biological relevance of a 50-node ADAGE model, and we expect that increasing the node number will allow for further separation of distinct processes with independent transcriptional signatures. Denoising autoencoders and other deep-learning based methods allow for a stacked representation that maps well to layers of biological regulation. Future work will focus on building larger and deeper networks to better model complex biological systems and on incorporating multiple data types into a single model. As reviewed by leaders in the field, unsupervised use is likely to be the future of deep learning (57), and we anticipate that ADAGE and other unsupervised deep-learning-based approaches will continue to complement traditional feature extraction methods, such as PCA and ICA, in this context. We anticipate that the collection of new data, particularly data measuring new environments and genetic perturbations, will continue to refine and improve the *P. aeruginosa* ADAGE model over time.

## MATERIALS AND METHODS

### Construction of a gene expression compendium for *P. aeruginosa*.

We downloaded a complete collection of *P. aeruginosa* gene expression data sets measured on the Affymetrix platform GPL84 with available supplemental CEL files from the ArrayExpress archive of the Functional Genomics Data (21) on 22 February 2014. This resulted in a collection of 109 distinct data sets covering 950 individual samples with measurements for 5,549 genes. We first combined these samples generated by different laboratories into one large expression compendium by using the rma function with quantile normalization provided in Bioconductor’s affy package in R (58); the resulting expression measurements are reported on a log_2_ scale. For autoencoder construction, we linearly transformed the expression range of each gene to be between 0 and 1. Validation data sets from the *Pseudomonas* GeneChip platform were processed concurrently through the rma function and linearly zero-one normalized using the same expression range as the compendium.

For RNA-Seq data sets, we retained genes intersecting with those existing in the compendium. The expression values of genes contained in the compendium but not measured by RNA-Seq were set to zero. To address the dynamic range differences between microarray and RNA-Seq platform analyses, we applied the training distribution matching (TDM) method to normalize RNA-Seq data and make them comparable to microarray data (42). As with the microarray validation sets, a linear zero-one normalization was performed after TDM.

### Training the ADAGE model.

We constructed a denoising autoencoder to summarize the *P. aeruginosa* gene expression compendium, which covers diverse genetic and environmental perturbations. We used the Theano (59) python library to implement DA training. To train one sample, we randomly corrupted a percentage of the genes (termed the corruption level) by setting their input values to 0 (18). The corrupted sample *x* serves as input to the DA. By multiplying the corrupted sample *x* by the weight matrix *W*, we calculated the activity vector *A* (equation 1). This activity vector represented the activities of each hidden node without considering the hidden bias vector *b* or the sigmoid transformation. To calculate the hidden representation *y*, we added the activity vector to *b* and applied a sigmoid transformation (equation 2). Next, we computed the reconstructed input *z* by multiplying *y* by the transpose of the weight matrix *W*′ and adding visible bias vector *b*′ (equation 3). Accurately reconstructing the input value thus represented a problem of fitting appropriate weight matrix and bias vectors to minimize the cross-entropy *L_H_* value between the initial input and the reconstructed input (equation 4). To accelerate the training process, we trained the DA in batches of samples, and the number of samples in each batch was termed the batch size. The reconstruction error was optimized through stochastic gradient descent with the weight matrix *W* and bias vectors *b* and *b*′ being updated in each batch. The magnitudes of weight and bias changes were controlled by a specified learning rate. Training proceeded through epochs, and in each epoch training used sufficient batches to include all training samples. Training stopped once the specified number of epochs (termed the epoch size) was reached. A detailed description of training for denoising autoencoders has been described by Vincent et al. (18).

(1) A=Wx

(2) Y=s(A+b)

(3) z=s(W'y+b')

(4) $$L_{H}\left(x,z\right)=-\sum_{k=1}^{d}\left[x_{k}\log z_{k}+\left(1-x_{k}\right)\log\left(1-z_{k}\right)\right]$$

To allow the manual interpretation of nodes, we fixed the number of nodes at 50 and named them “node ##” based on the order in which they appeared. We used the parameters identified as suitable for a gene expression compendium by Tan et al. (60): a batch size of 10 over 500 training epochs with a corruption level of 0.1 and a learning rate of 0.01.

After the DA was fully trained and the weight matrix was fixed, we calculated the activity value for each specific node for each specific sample in the training pool by computing the dot product of the row vector for that node in the weight matrix and the gene expression vector of the sample. We calculated the activity values of samples in newly generated validation experiments, which were not included in the training pool, in the same manner.

### Identification of high-weight genes for each ADAGE node.

Each gene was connected to each node through a value in the weight matrix, *W*. For each node, this learned vector of weights connected that node to each gene. We calculated the standard deviation of each node’s weight vector and defined a set of HW genes for the node that had weights 2 or more standard deviations away from the mean. This set of HW genes summarized the genes with the strongest influence on the node’s activity.

### Association of *P. aeruginosa* operons with specific ADAGE nodes.

To associate a specific set of operons to a node, we carried out a Gene Set Enrichment Analysis (GSEA) (46) for weight vectors. The weight vector corresponding to each constructed node was used as the weighted gene list. The curated operon information was downloaded from the Database of prOkaryotic OpeRons (DOOR) (24). We considered operons consisting of three or more genes as potential target gene sets in GSEA, and we used a false discovery rate threshold of 0.05 to identify significant associations. We calculated the overall coverage of operons as the ratio of the number of operons significantly associated with at least one node to the total number of operons curated in DOOR. Operons significantly associated with each node are provided in File S4 in the supplemental material.

### Evaluation of the association between gene positions and ADAGE weights.

Bacterial genes are grouped by function in the genome. We tested the ADAGE model’s ability to capture such relationships in the learned weight matrix. We fitted a logistic regression model (equation 5) with the goal of predicting whether or not a gene would be HW for a node based on two factors: the number of genes between the pair of genes (*d*) and whether or not it is cooperonic with an HW gene in the same node (*c*). We also included an interaction term between *d* and *c* in the model. We considered *d* values in the range from 1 to 10 and disregarded genes that were more than 10 genes away from the gene in question. We tested the significance of the coefficients on *d*, *c*, and the interaction term to assess the extent to which each indicated a relationship with a gene’s likelihood of being *HW* in a node.

(5) HW=d+c+d×c

### Gene function assignment with the ADAGE weight matrix.

We assessed the extent to which the ADAGE model captured the genes’ functions. We employed a simple 1-nearest-neighbor classifier to assign the function of a gene. Each gene was connected to nodes through a weight vector of length 50  (for a 50-node ADAGE model). For a target gene, we calculated the Euclidean distance between that gene’s weight vector and the weight vectors of all other genes. We considered the nearest neighbor to be the gene with the shortest distance. We assigned the KEGG function or functions of this closest neighbor to the target gene. To evaluate this assignment, we used KEGG pathways as the gold standards for gene function. Because one gene could be annotated with multiple KEGG pathways, we used two assessment criteria. In the first, we considered a function assignment to be correct as long as there existed an overlap between the assigned pathway and the gene’s annotated pathways. As a second evaluation, we used a more stringent definition of a correct assignment that required all of the predicted and annotated pathways to match. To evaluate the extent to which the ADAGE weight matrix captured gene functions, we compared observed accuracies with the performance of 1,000 weight matrices with randomly permuted gene labels. These matrices preserve the overall weight distributions for each node but eliminate the relationship between genes and their weights. The distribution of the prediction accuracy using permuted weight matrices was plotted.

### Analysis of sequence divergence and gene expression using Affymetrix *P. aeruginosa* GeneChips.

To compare *P. aeruginosa* wild-type and *Δanr* strains on airway epithelial cells, *P. aeruginosa* biofilms were grown on airway epithelial cells homozygous for the *CFTR*ΔF508 mutation (CFBE41o^−^) (61) as described previously (43, 62). Data were comprised of 3 biological replicates each for wild-type PAO1 and the Δ*anr* mutant. Briefly, stationary-phase cultures of *P. aeruginosa* grown in LB shaken at 37°C were washed twice and resuspended in minimal essential medium (MEM) at an OD_600_ of 0.5. These suspensions were applied to confluent CFBE41o^−^ cells grown in 24-well plastic dishes (MatTek Corp., Ashland, MA) and incubated at 37°C, 5% CO_2_ for 1 h, after which planktonic cells were aspirated away and medium was replaced. After an additional 90 min, planktonic cells were removed again and the monolayer was washed twice with phosphate-buffered saline (PBS). Epithelial cells and attached bacterial biofilms were treated with lysozyme, and RNA was harvested using an RNeasy kit (Qiagen). RNA samples were treated with RQ1 DNase from Promega to remove contaminating DNA, and a MICROBExpress bacterial mRNA enrichment kit (Life Technologies) was used to deplete eukaryotic RNA from the samples.

For each RNA sample, cDNA samples were synthesized with SuperScript III reverse transcriptase (Invitrogen, Carlsbad, CA) and NS5 primers instead of random hexamers. The cDNAs were terminally labeled with biotin-ddUTP (Enzo Bio-Array terminal labeling kit; Affymetrix) and hybridized to Affymetrix *Pseudomonas* GeneChips according to the manufacturer’s instructions with the GeneChip fluidics station 450 (Affymetrix). GeneChips were scanned with the GeneChip Scanner 3000 7G (Affymetrix) in the Dartmouth Genomics Shared Resource laboratory. The BioConductor Affy library was used to process CEL files as described above for the compendium. 

For the genome hybridization analysis of *P. aeruginosa* strains PA14 and PAO1, genomic DNA was isolated, digested using DNase I, denatured at 100°C for 10 min, and then labeled as described above. The GeneChips were processed as described above.

### Node interpretation with GO and KEGG.

We used the experimentally derived annotations from Gene Ontology (GO) (29, 63) and KEGG pathways (25) of *P. aeruginosa* to identify the biological features captured by each node. Only terms that had more than 5 genes but fewer than 100 genes were considered. We calculated an odds ratio that indicated how overrepresented each GO/KEGG term was in each node’s HW genes. The top 10 enriched pathways for some selected nodes are listed in Table S1 in the supplemental material, and the full list for all 50 nodes can be downloaded from the online repository (https://github.com/greenelab/adage/blob/7a4eda39d360b224268921dc1f2c14b32788ab16/Node_interpretation/GO_KEGG_enrichment.txt).

### Sequence alignment and comparison across strains.

The DNA sequences of 13 strains that have been sequenced before were obtained from the *Pseudomonas* Genome Database (64). Orthologous genes across 13 strains were aligned using Clustal Omega (65) via the EMBL-EBI webserver (66), and the alignment results, including percent identity matrices and phylogenetic trees, were downloaded. The phylogenetic trees in Fig. 3D were drawn using tree graph 2 (67).

### Principal component analysis and independent component analysis.

PCA and ICA were performed in R using the prcomp function and fastICA function from the fastICA package (68). For PCA, we used the matrix of variable loadings as an analog to ADAGE’s weight matrix. For ICA, we used the product of the prewhitening matrix and the estimated unmixing matrix as the weight matrix, which first projects data onto the first 50 principal components and then projects them onto the independent components. HW genes for each component were defined in the same manner as with ADAGE: genes outside 2 standard deviations of each method’s weight distribution.

### ADAGE model and source code availability.

To facilitate the use of ADAGE by the *P. aeruginosa* research community, we have generated an ADAGE analysis of all of the publicly available *P. aeruginosa* gene expression experiments included in our compendium (see File S5 in the supplemental material) and provide open source code to perform construction of ADAGE models and their application to newly generated data (https://github.com/greenelab/adage).

### Microarray data accession numbers.

Data from the genome hybridization experiment have been uploaded to GEO and are available under accession number GSE67038. The data obtained from the wild-type and Δ*anr* strains on airway epithelial cells have been uploaded to GEO and are available under accession number GSE67006.
