# Supplementary material for: ADAGE-Based Integration of Publicly Available Pseudomonas aeruginosa Gene Expression Data with Denoising Autoencoders Illuminates Microbe-Host Interactions
Source: mSystems. 2016 Jan 19;1(1):e00025-15. doi: 10.1128/mSystems.00025-15 (PMC5069748; doi:10.1128/mSystems.00025-15)
Supplement: Table S1 [file sys001160033st6.docx]

**Supplemental Table 1**: Top 10 associated GO terms and KEGG pathways for each node mentioned in the paper.

| Node | GO terms | Odds Ratio | KEGG terms | Odds Ratio |
| --- | --- | --- | --- | --- |
| Node 16 | GO-BP-0007155:cell adhesion | 16.01 | KEGG-Pathway-pae01053: Biosynthesis of siderophore group nonribosomal peptides - Pseudomonas aeruginosa PAO1 | 42.883 |
| Node 16 | GO-BP-0006536:glutamate metabolic process | 14.177 | KEGG-Module-M00529: Denitrification, nitrate => nitrogen | 32.412 |
| Node 16 | GO-BP-0043648:dicarboxylic acid metabolic process | 14.177 | KEGG-Module-M00328: Hemophore/metalloprotease transport system | 21.351 |
| Node 16 | GO-MF-0090484:drug transporter activity | 14.177 | KEGG-Module-M00018: Threonine biosynthesis, aspartate => homoserine => threonine | 21.351 |
| Node 16 | GO-BP-0032101:regulation of response to external stimulus | 14.177 | KEGG-Module-M00335: Sec (secretion) system | 17.927 |
| Node 16 | GO-BP-0022610:biological adhesion | 12.806 | KEGG-Pathway-pae00643: Styrene degradation - Pseudomonas aeruginosa PAO1 | 15.363 |
| Node 16 | GO-MF-0008233:peptidase activity | 10.669 | KEGG-Pathway-pae03060: Protein export - Pseudomonas aeruginosa PAO1 | 10.788 |
| Node 16 | GO-BP-0051248:negative regulation of protein metabolic process | 10.631 | KEGG-Module-M00530: Dissimilatory nitrate reduction, nitrate => ammonia | 7.084 |
| Node 16 | GO-BP-0072330:monocarboxylic acid biosynthetic process | 10.631 | KEGG-Pathway-pae00750: Vitamin B6 metabolism - Pseudomonas aeruginosa PAO1 | 6.071 |
| Node 16 | GO-MF-0016853:isomerase activity | 8.503 | KEGG-Module-M00156: Cytochrome c oxidase, cbb3-type | 6.071 |
| Node 25 | GO-CC-0016020:membrane | 8.467 | KEGG-Pathway-pae00471: D-Glutamine and D-glutamate metabolism - Pseudomonas aeruginosa PAO1 | 14.117 |
| Node 25 | GO-BP-0043107:type IV pilus-dependent motility | 6.556 | KEGG-Module-M00299: Spermidine/putrescine transport system | 10.663 |
| Node 25 | GO-BP-0032409:regulation of transporter activity | 5.272 | KEGG-Module-M00053: Pyrimidine deoxyribonuleotide biosynthesis, CDP/CTP => dCDP/dCTP,dTDP/dTTP | 10.624 |
| Node 25 | GO-CC-0031975:envelope | 5.272 | KEGG-Module-M00021: Cysteine biosynthesis, serine => cysteine | 10.586 |
| Node 25 | GO-BP-0046889:positive regulation of lipid biosynthetic process | 5.272 | KEGG-Module-M00307: Pyruvate oxidation, pyruvate => acetyl-CoA | 7.965 |
| Node 25 | GO-BP-0045834:positive regulation of lipid metabolic process | 5.272 | KEGG-Pathway-pae00520: Amino sugar and nucleotide sugar metabolism - Pseudomonas aeruginosa PAO1 | 7.204 |
| Node 25 | GO-BP-0009057:macromolecule catabolic process | 5.272 | KEGG-Pathway-pae00550: Peptidoglycan biosynthesis - Pseudomonas aeruginosa PAO1 | 7.128 |
| Node 25 | GO-CC-0030313:cell envelope | 5.272 | KEGG-Module-M00034: Methionine salvage pathway | 7.079 |
| Node 25 | GO-CC-0030312:external encapsulating structure | 5.272 | KEGG-Pathway-pae00052: Galactose metabolism - Pseudomonas aeruginosa PAO1 | 5.272 |
| Node 25 | GO-CC-0071944:cell periphery | 5.272 | KEGG-Module-M00644: Multidrug resistance, efflux pump MexHI-OprD | 5.272 |
| Node 29 | GO-MF-0016874:ligase activity | 25.635 | KEGG-Module-M00157: F-type ATPase, prokaryotes and chloroplasts | 32.161 |
| Node 29 | GO-BP-0008300:isoprenoid catabolic process | 14.365 | KEGG-Module-M00144: NADH:quinone oxidoreductase, prokaryotes | 19.424 |
| Node 29 | GO-BP-0044242:cellular lipid catabolic process | 11.489 | KEGG-Pathway-pae00471: D-Glutamine and D-glutamate metabolism - Pseudomonas aeruginosa PAO1 | 12.725 |
| Node 29 | GO-BP-0016042:lipid catabolic process | 11.489 | KEGG-Module-M00011: Citrate cycle, second carbon oxidation, 2-oxoglutarate => oxaloacetate | 10.548 |
| Node 29 | GO-BP-0006720:isoprenoid metabolic process | 11.489 | KEGG-Pathway-pae00550: Peptidoglycan biosynthesis - Pseudomonas aeruginosa PAO1 | 10.447 |
| Node 29 | GO-MF-0016835:carbon-oxygen lyase activity | 9.542 | KEGG-Pathway-pae00190: Oxidative phosphorylation - Pseudomonas aeruginosa PAO1 | 8.97 |
| Node 29 | GO-MF-0016836:hydro-lyase activity | 9.542 | KEGG-Module-M00529: Denitrification, nitrate => nitrogen | 8.204 |
| Node 29 | GO-BP-1901293:nucleoside phosphate biosynthetic process | 9.542 | KEGG-Module-M00036: Leucine degradation, leucine => acetoacetate + acetyl-CoA | 7.408 |
| Node 29 | GO-BP-0009991:response to extracellular stimulus | 9.542 | KEGG-Module-M00009: Citrate cycle (TCA cycle, Krebs cycle) | 6.817 |
| Node 29 | GO-BP-0009267:cellular response to starvation | 9.542 | KEGG-Module-M00300: Putrescine transport system | 6.359 |
| Node 30 | GO-BP-0007155:cell adhesion | 9.541 | KEGG-Module-M00529: Denitrification, nitrate => nitrogen | 16.042 |
| Node 30 | GO-BP-0043683:type IV pilus biogenesis | 8.98 | KEGG-Module-M00222: Phosphate transport system | 15.907 |
| Node 30 | GO-BP-0050709:negative regulation of protein secretion | 7.98 | KEGG-Module-M00021: Cysteine biosynthesis, serine => cysteine | 11.928 |
| Node 30 | GO-BP-0051048:negative regulation of secretion | 7.98 | KEGG-Module-M00324: Dipeptide transport system | 9.541 |
| Node 30 | GO-BP-0051224:negative regulation of protein transport | 7.98 | KEGG-Module-M00332: Type III secretion system | 9.285 |
| Node 30 | GO-BP-0043711:pilus organization | 7.98 | KEGG-Module-M00122: Cobalamin biosynthesis, cobinamide => cobalamin | 6.812 |
| Node 30 | GO-BP-0009297:pilus assembly | 7.98 | KEGG-Pathway-pae00520: Amino sugar and nucleotide sugar metabolism - Pseudomonas aeruginosa PAO1 | 5.583 |
| Node 30 | GO-BP-0022610:biological adhesion | 7.949 | KEGG-Module-M00188: NitT/TauT family transport system | 4.332 |
| Node 30 | GO-BP-0043107:type IV pilus-dependent motility | 7.394 | KEGG-Pathway-pae02040: Flagellar assembly - Pseudomonas aeruginosa PAO1 | 4.011 |
| Node 30 | GO-BP-0030031:cell projection assembly | 5.982 | KEGG-Module-M00124: Pyridoxal biosynthesis, erythrose-4P => pyridoxal-5P | 3.957 |
| Node 33 | GO-MF-0005515:protein binding | 26.786 | KEGG-Module-M00436: Sulfonate transport system | 36.318 |
| Node 33 | GO-BP-0009057:macromolecule catabolic process | 11.862 | KEGG-Module-M00223: Phosphonate transport system | 23.886 |
| Node 33 | GO-BP-0050709:negative regulation of protein secretion | 8.949 | KEGG-Pathway-pae00920: Sulfur metabolism - Pseudomonas aeruginosa PAO1 | 11.149 |
| Node 33 | GO-BP-0051048:negative regulation of secretion | 8.949 | KEGG-Pathway-pae00440: Phosphonate and phosphinate metabolism - Pseudomonas aeruginosa PAO1 | 10.229 |
| Node 33 | GO-BP-0051224:negative regulation of protein transport | 8.949 | KEGG-Pathway-pae00562: Inositol phosphate metabolism - Pseudomonas aeruginosa PAO1 | 7.115 |
| Node 33 | GO-BP-0030254:protein secretion by the type III secretion system | 8.922 | KEGG-Module-M00237: Branched-chain amino acid transport system | 6.689 |
| Node 33 | GO-BP-0051248:negative regulation of protein metabolic process | 8.895 | KEGG-Module-M00126: Tetrahydrofolate biosynthesis, GTP => THF | 5.944 |
| Node 33 | GO-BP-0071241:cellular response to inorganic substance | 8.895 | KEGG-Pathway-pae00740: Riboflavin metabolism - Pseudomonas aeruginosa PAO1 | 5.08 |
| Node 33 | GO-BP-0007155:cell adhesion | 7.115 | KEGG-Pathway-pae00430: Taurine and hypotaurine metabolism - Pseudomonas aeruginosa PAO1 | 5.08 |
| Node 33 | GO-BP-0044092:negative regulation of molecular function | 7.115 | KEGG-Pathway-pae00450: Selenocompound metabolism - Pseudomonas aeruginosa PAO1 | 4.444 |
| Node 39 | GO-BP-0030254:protein secretion by the type III secretion system | 63.362 | KEGG-Module-M00332: Type III secretion system | 318.811 |
| Node 39 | GO-MF-0005515:protein binding | 26.786 | KEGG-Module-M00324: Dipeptide transport system | 13.388 |
| Node 39 | GO-BP-0050709:negative regulation of protein secretion | 18.027 | KEGG-Pathway-pae00680: Methane metabolism - Pseudomonas aeruginosa PAO1 | 4.479 |
| Node 39 | GO-BP-0051048:negative regulation of secretion | 18.027 | KEGG-Pathway-pae00401: Novobiocin biosynthesis - Pseudomonas aeruginosa PAO1 | 4.432 |
| Node 39 | GO-BP-0051224:negative regulation of protein transport | 18.027 | KEGG-Module-M00190: Iron(III) transport system | 4.432 |
| Node 39 | GO-BP-0045184:establishment of protein localization | 11.76 | KEGG-Module-M00185: Sulfate transport system | 4.432 |
| Node 39 | GO-BP-0015031:protein transport | 11.76 | KEGG-Module-M00235: Arginine/ornithine transport system | 4.432 |
| Node 39 | GO-BP-0008104:protein localization | 11.76 | KEGG-Pathway-pae00460: Cyanoamino acid metabolism - Pseudomonas aeruginosa PAO1 | 3.95 |
| Node 39 | GO-BP-0060341:regulation of cellular localization | 10.808 | KEGG-Module-M00237: Branched-chain amino acid transport system | 3.95 |
| Node 39 | GO-BP-0050708:regulation of protein secretion | 10.808 | KEGG-Pathway-pae02040: Flagellar assembly - Pseudomonas aeruginosa PAO1 | 3.713 |
| Node 42 | GO-BP-0006644:phospholipid metabolic process | 19.304 | KEGG-Module-M00529: Denitrification, nitrate => nitrogen | 12.909 |
| Node 42 | GO-BP-0007155:cell adhesion | 14.475 | KEGG-Module-M00156: Cytochrome c oxidase, cbb3-type | 9.647 |
| Node 42 | GO-MF-0022891:substrate-specific transmembrane transporter activity | 12.909 | KEGG-Pathway-pae00562: Inositol phosphate metabolism - Pseudomonas aeruginosa PAO1 | 7.691 |
| Node 42 | GO-BP-0006527:arginine catabolic process | 12.909 | KEGG-Module-M00258: Putative ABC transport system | 6.408 |
| Node 42 | GO-MF-0015075:ion transmembrane transporter activity | 12.909 | KEGG-Module-M00036: Leucine degradation, leucine => acetoacetate + acetyl-CoA | 5.524 |
| Node 42 | GO-MF-0008324:cation transmembrane transporter activity | 12.909 | KEGG-Pathway-pae00072: Synthesis and degradation of ketone bodies - Pseudomonas aeruginosa PAO1 | 4.804 |
| Node 42 | GO-MF-0009055:electron carrier activity | 12.909 | KEGG-Module-M00200: Putative sorbitol/mannitol transport system | 4.79 |
| Node 42 | GO-BP-0009066:aspartate family amino acid metabolic process | 12.822 | KEGG-Pathway-pae00280: Valine, leucine and isoleucine degradation - Pseudomonas aeruginosa PAO1 | 4.006 |
| Node 42 | GO-CC-0031975:envelope | 12.822 | KEGG-Module-M00242: Zinc transport system | 3.831 |
| Node 42 | GO-BP-0010043:response to zinc ion | 12.822 | KEGG-Module-M00018: Threonine biosynthesis, aspartate => homoserine => threonine | 3.831 |
