# Supplementary material for: ADAGE-Based Integration of Publicly Available Pseudomonas aeruginosa Gene Expression Data with Denoising Autoencoders Illuminates Microbe-Host Interactions
Source: mSystems. 2016 Jan 19;1(1):e00025-15. doi: 10.1128/mSystems.00025-15 (PMC5069748; doi:10.1128/mSystems.00025-15)
Supplement: Table S2 [file sys001160033st7.docx]

**Supplemental Table 2**: Anr-regulated gene list used to identify nodes significantly enriched of genes regulated by Anr.

| **PA No.** | **Gene** |
| --- | --- |
| PA5475 |  |
| PA1673 |  |
| PA5027 |  |
| PA3337 | rfaD |
| PA4348 |  |
| PA4347 |  |
| PA4346 |  |
| PA0527 | dnr |
| PA2119 |  |
| PA0200 |  |
| PA0519 | nirS |
| PA0518 | nirM |
| PA0517 | nirC |
| PA0516 | nirF |
| PA0515 |  |
| PA0514 | nirL |
| PA0513 |  |
| PA0512 |  |
| PA0511 | nirJ |
| PA0510 |  |
| PA0509 | nirN |
| PA1546 | hemN |
| PA5232 |  |
| PA5231 |  |
| PA5230 |  |
| PA4577 |  |
| PA0141 |  |
| PA1746 |  |
| PA1557 | ccoN2 |
| PA1556 | ccoO2 |
| PA1555 | ccoP2 |
| PA1554 | ccoN1 |
| PA4067 | oprG |
| PA5427 | adhA |
| PA4352 |  |
| PA2127 |  |
| PA2126 |  |
| PA2125 |  |
| PA3930 | cioA |
| PA3929 | cioB |
| PA3928 |  |
| PA4587 | ccpR |
| PA0024 | hemF |
| PA0459 |  |
| PA0520 | nirQ |
| PA0521 |  |
| PA0522 |  |
| PA0523 | norC |
| PA0524 | norB |
| PA0525 |  |
| PA0526 |  |
| PA0836 | ackA |
| PA1561 | aer |
| PA1789 |  |
| PA1863 | modA |
| PA1862 | modB |
| PA1861 | modC |
| PA2193 | hcnA |
| PA2194 | hcnB |
| PA2195 | hcnC |
| PA3190 |  |
| PA3309 |  |
| PA3391 | nosR |
| PA3877 | narK1 |
| PA3876 | narK2 |
| PA3878 | narX |
| PA3879 | narL |
| PA4236 | katA |
| PA4328 |  |
| PA4922 | azu |
| PA5170 | arcD |
| PA5171 | arcA |
