# Supplementary figures and images for: ADAGE-Based Integration of Publicly Available Pseudomonas aeruginosa Gene Expression Data with Denoising Autoencoders Illuminates Microbe-Host Interactions
Source: mSystems. 2016 Jan 19;1(1):e00025-15. doi: 10.1128/mSystems.00025-15 (PMC5069748; doi:10.1128/mSystems.00025-15)

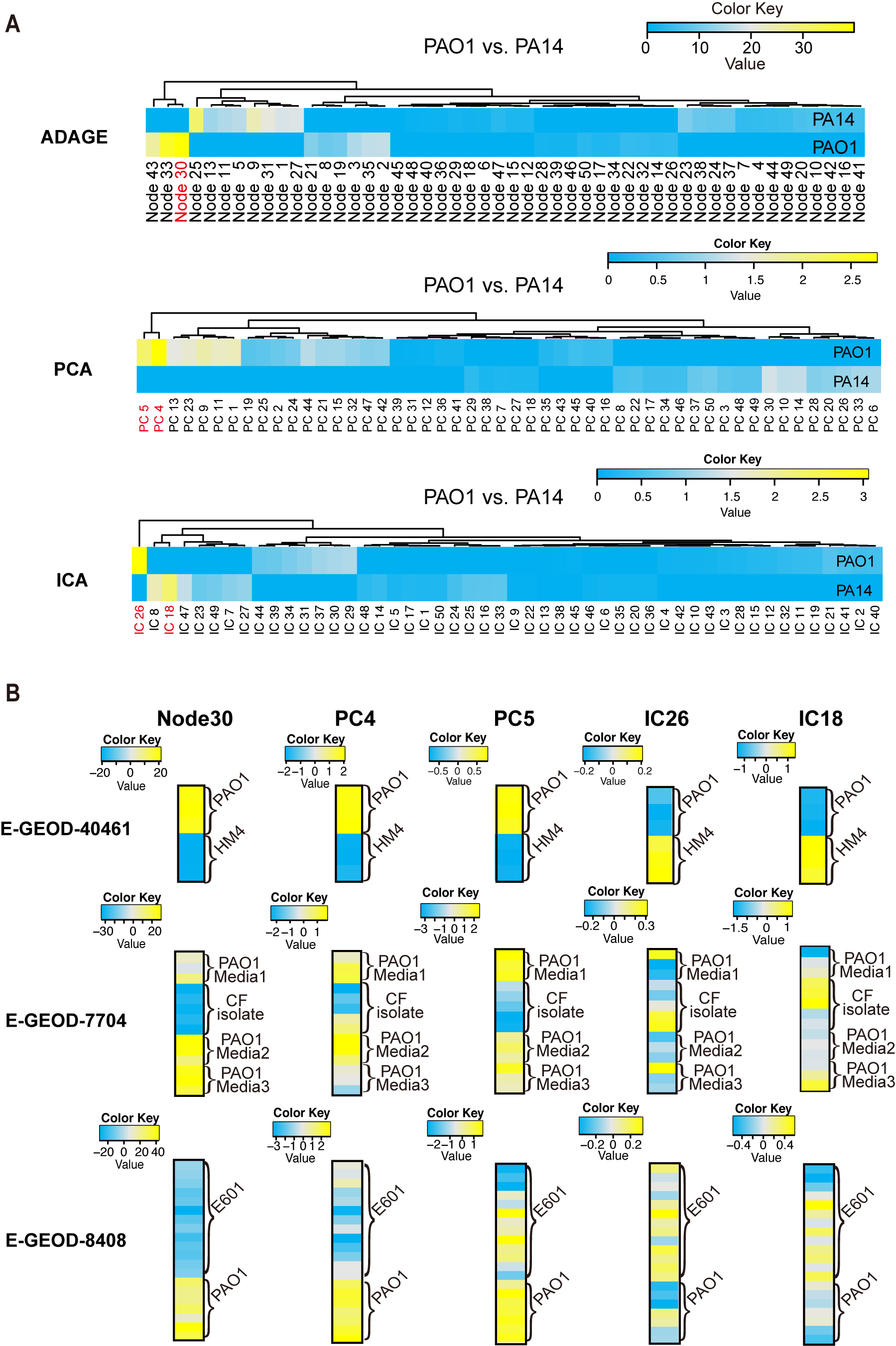

Supplement: Figure S1 [file sys001160033sf8.tif]

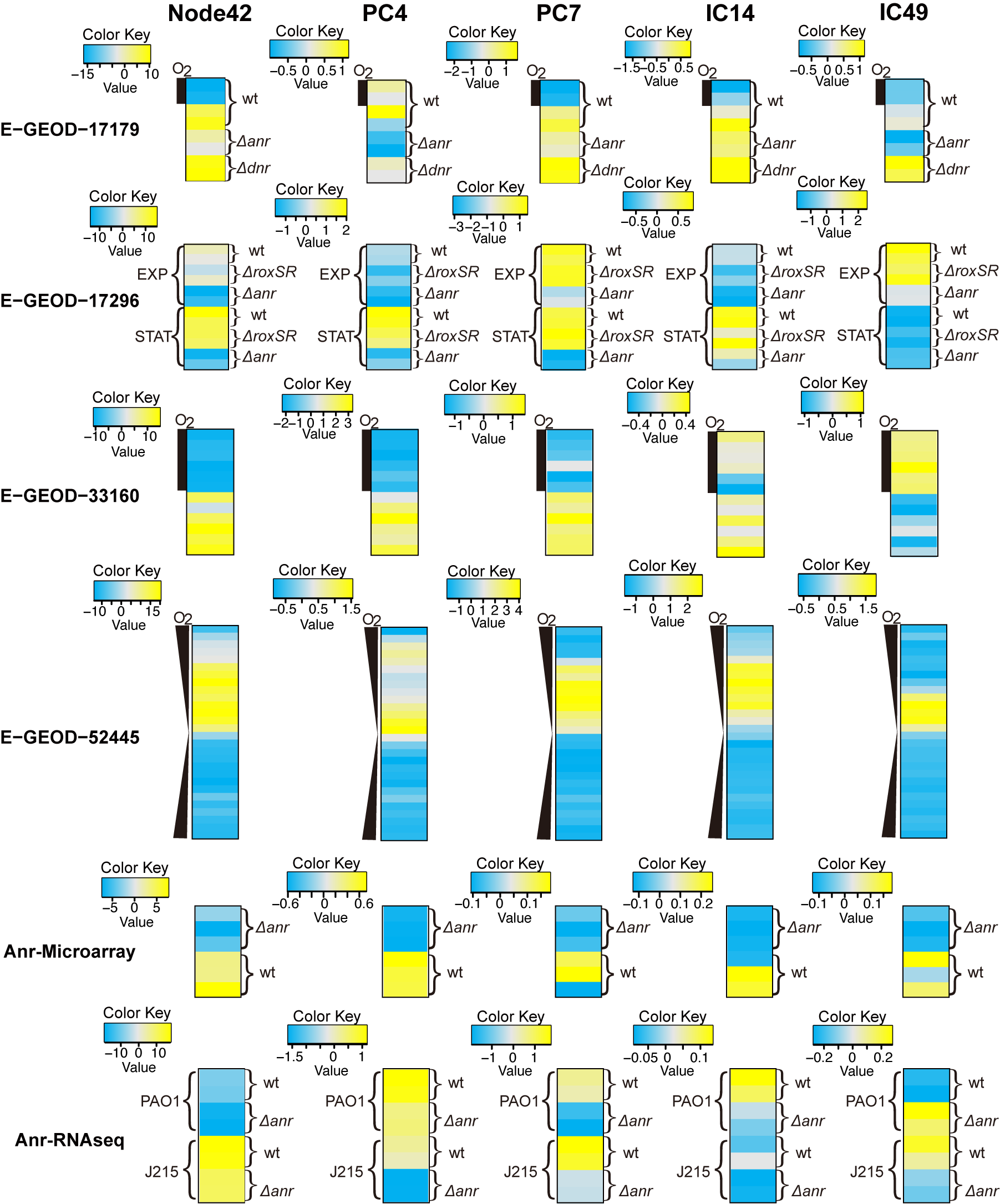

Supplement: Figure S2 [file sys001160033sf9.tif]

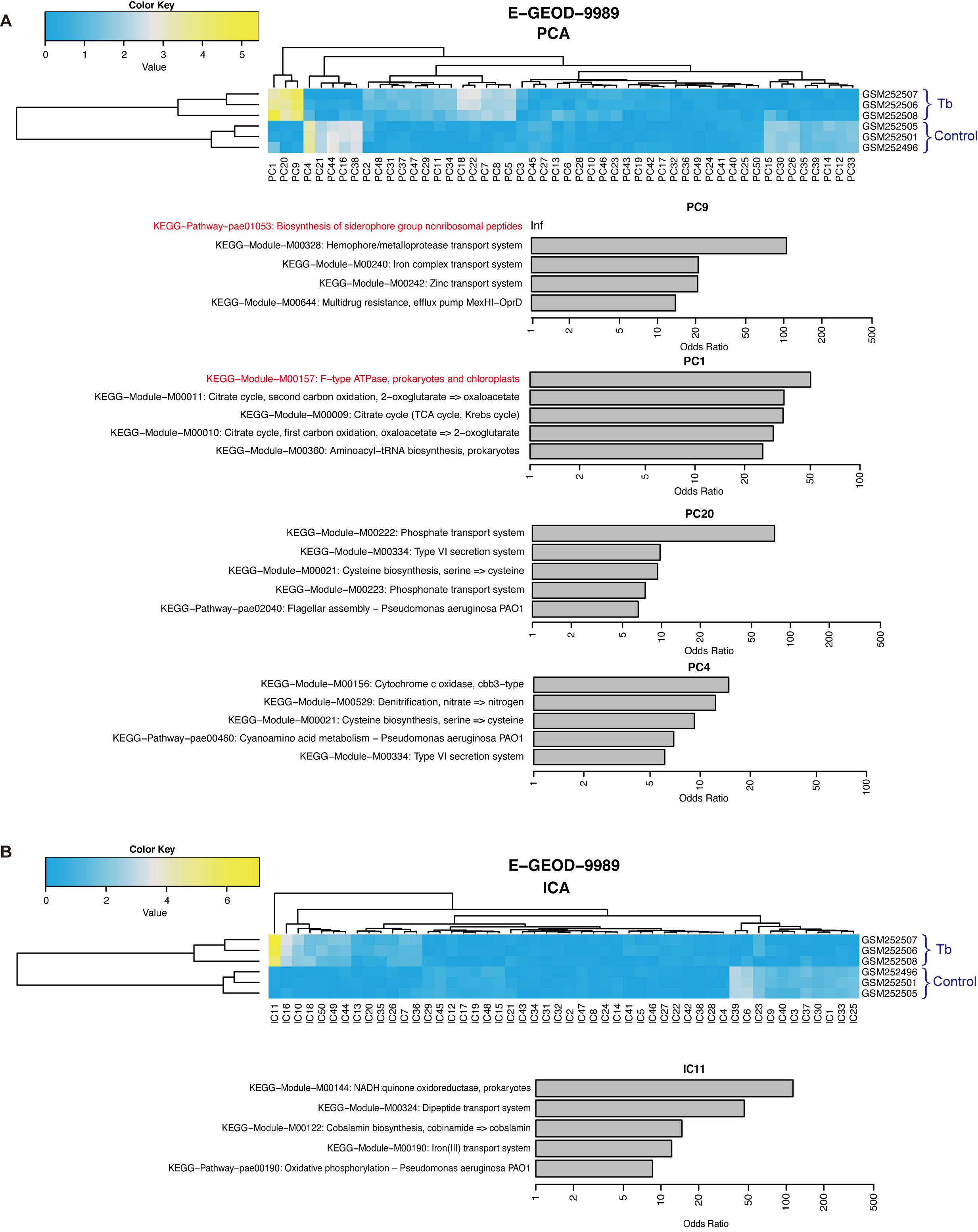

Supplement: Figure S3 [file sys001160033sf10.tif]
